# Brain Temperature as an Indicator of Cognitive Function in Traumatic Brain Injury Patients

**DOI:** 10.3390/metabo14010017

**Published:** 2023-12-27

**Authors:** Maho Kitagawa, Kagari Abiko, Sulaiman Sheriff, Andrew A. Maudsley, Xinnan Li, Daisuke Sawamura, Sinyeob Ahn, Khin Khin Tha

**Affiliations:** 1 Laboratory for Biomarker Imaging Science, Graduate School of Biomedical Science and Engineering, Hokkaido University, N15 W7, Kita-ku, Sapporo 060-8638, Japan; kitagawa846@eis.hokudai.ac.jp (M.K.); lixinnan820@yahoo.co.jp (X.L.); 2 Department of Rehabilitation, Hokkaido University Hospital, Sapporo 060-8648, Japan; kagari.abiko@gmail.com; 3 Department of Rehabilitation, Sapporo Azabu Neurosurgical Hospital, Sapporo 065-0022, Japan; 4 Department of Radiology, University of Miami Miller School of Medicine, Miami, FL 33136, USA; ssheriff@med.miami.edu (S.S.); amaudsley@med.miami.edu (A.A.M.); 5 Department of Rehabilitation Science, Hokkaido University Faculty of Health Sciences, Sapporo 060-0812, Japan; d.sawamura@pop.med.hokudai.ac.jp; 6 Siemens Healthineers, San Francisco, CA 94553, USA; sinyeob.ahn@siemens-healthineers.com; 7 Global Center for Biomedical Science and Engineering, Faculty of Medicine, Hokkaido University, N15 W7, Kita-ku, Sapporo 060-8638, Japan

**Keywords:** traumatic brain injury, echo-planar, magnetic resonance spectroscopic imaging, metabolite, whole-brain, temperature

## Abstract

Whether brain temperature noninvasively extracted by magnetic resonance imaging has a role in identifying brain changes in the later phases of mild to moderate traumatic brain injury (TBI) is not known. This prospective study aimed to evaluate if TBI patients in subacute and chronic phases had altered brain temperature measured by whole-brain magnetic resonance spectroscopic imaging (WB-MRSI) and if the measurable brain temperature had any relationship with cognitive function scores. WB-MRSI was performed on eight TBI patients and fifteen age- and sex-matched control subjects. Brain temperature (T) was extracted from the brain’s major metabolites and compared between the two groups. The T of the patients was tested for correlation with cognitive function test scores. The results showed significantly lower brain temperature in the TBI patients (*p* < 0.05). Brain temperature derived from N-acetylaspartate (T_NAA_) strongly correlated with the 2 s paced auditory serial addition test (PASAT-2s) score (*p* < 0.05). The observation of lower brain temperature in TBI patients may be due to decreased metabolic activity resulting from glucose and oxygen depletion. The correlation of brain temperature with PASAT-2s may imply that noninvasive brain temperature may become a noninvasive index reflecting cognitive performance.

## 1. Introduction

Traumatic brain injury (TBI) is a brain injury caused by an external force, such as falls and vehicle collisions [1]. About 69 million people are globally affected annually [2]. The direct cost of TBI can amount to USD 13.1 billion. The indirect cost of TBI is even more enormous; it is reported that the cost due to loss of productivity amounts to USD 64.7 billion [3]. Often, the burden of total cost is initially underestimated since cognitive impairment in mild TBI is left unnoticed until late. Of mild, moderate, and severe TBI classified based on the level of consciousness or the Glasgow Coma Scale (GCS) at the time of initial hospital admission [4,5], the outcome of severe TBI is often recognized in the early phases of injury. Yet, the burden posed by the consequences of mild and moderate TBI cannot be neglected. According to a previous survey, one-third of mild TBI and two-thirds of moderate TBI patients remain unemployed three months after injury [6,7,8]. Impaired memory, attention, processing speed, and executive function have been reported in mild and moderate TBI patients in subacute (8 to 89 days from injury) and chronic (after 90 days) phases [9,10,11].

Rehabilitation proves to be effective in improving cognitive function after brain injury [12]. To correctly estimate and reduce the socioeconomic burden of TBI, mild to moderate TBI patients must receive appropriate cognitive assessment and rehabilitation as early as possible. However, completing the lengthy cognitive assessment protocols is not always easy when ill patients cannot cooperate. Thus, patients’ cognitive status needs to be assessed through alternate means that do not demand patient cooperation. 

Computed tomography (CT) and magnetic resonance imaging (MRI) are typically used to diagnose TBI [9,13,14]. MRI is particularly effective in identifying brain injuries such as cerebral contusion and diffuse axonal injury (DAI), owing to its superior tissue contrast [15]. With new quantitative MRI techniques, minute abnormalities at the microstructure level can even be identified [16,17]. These quantitative MRI techniques not only detect structural abnormalities but also aberrations in the brain’s metabolism. Proton magnetic resonance spectroscopy (^1^H-MRS), a quantitative MRI technique, has successfully identified areas with abnormal brain major metabolite ratios, such as N-acetylaspartate (NAA)/creatine (Cr) and choline (Cho)/Cr, in otherwise normal-appearing brain tissue of mild TBI patients [18]. More importantly, a prior study has shown the correlation of these metabolite changes in certain brain regions with the cognitive status of TBI patients [19]. 

Areas of brain involvement usually differ among TBI patients. While area-wise assessment of the brain can provide estimates of cognitive involvement, a singular quantitative index applicable to all brain regions would allow us to compare severity across patients. Brain temperature, reflective of the brain’s energy consumption, can be a good candidate for this. Prior research has reported changes in the brain temperature’s circadian rhythm in acute (within 7 days from injury) moderate to severe TBI patients and the magnitude of this alteration as a predictor of survival [20]. Although the conventional way of measuring brain temperature involves inserting a thermistor into the brain parenchyma, less invasive alternatives have also been introduced. Brain temperature measurement by ^1^H-MRS is among these alternatives. This technique calculates the brain temperature from the frequency or amplitude of major brain metabolites [21,22]. The accuracy of the measurement has also been documented through phantom and animal experiments [23,24]. Its clinical utility has been reported in several pathological states, including myalgic encephalomyelitis/chronic fatigue syndrome [25]. 

To our knowledge, no brain temperature documentation about TBI in the late phases exists. We hypothesized that the brain temperature measured by ^1^H-MRS would be altered in these patients, which can indirectly reflect cognitive function. This prospective study aimed to evaluate if TBI patients in subacute and chronic phases had altered brain temperature measured by ^1^H-MRS and if the brain temperature measured by ^1^H-MRS had any relationship with cognitive function scores. 

## 2. Materials and Methods

### 2.1. Participants

The institutional review board of Hokkaido University Hospital, Sapporo, Japan, approved this prospective study (015-0271). Written informed consent was obtained from all participants. TBI patients in subacute to chronic phase who consulted at the Department of Rehabilitation, Hokkaido University Hospital, and consented to participate in this study were recruited over 25 months (November 2015–December 2017). The inclusion criteria were (1) male sex, (2) 20 years of age or older, and (3) diagnosed to have neurological sequelae of brain injury. The exclusion criteria were (1) absolute contraindications to MRI, (2) lack of MRI including short echo time (TE) whole-brain magnetic resonance spectroscopic imaging (WB-MRSI), (3) image artifacts that limit interpretation, (4) lack of cognitive function test results, and (5) concurrent central nervous system (CNS) pathologies either reported or detected on conventional MRI sequences. Eleven patients consented to this study. Of them, three were excluded for lacking short TE WB-MRSI. Eight patients (mean age ± standard deviation (SD) = 44.00 ± 13.34 years; age range = 20–68 years) were thus eligible for the study. The demographic details of the patients are provided in Table 1. Five of them were diagnosed with mild TBI, and three with moderate to severe TBI. The average interval ± SD between the injury and MRI was 73.63 ± 136.91 months (range = 2–432 months). 

As the control group, 15 age-matched males (age range = 22–68 years) were selected from a WB-MRSI database of 21 male and 17 female healthy adults [26]. The inclusion criteria for the normal database were (1) male sex and (2) age 20 years or older. The exclusion criteria were (1) contraindications to MRI, (2) history of diseases that affect the integrity of the CNS, and (3) visible motion artifacts. 

A radiologist with 22 years of experience in neuroimaging reviewed the images of conventional MRI sequences of the patients to exclude any gross abnormalities and image artifacts. 

### 2.2. MRI

All examinations were conducted using a 3T scanner (MAGNETOM Prisma, Siemens Healthcare, Erlangen, Germany) and a 64-channel head/neck coil. 

In all participants, WB-MRSI was performed using an echo-planar sequence and the following scan parameters: repetition time (TR)/TE/inversion time (TI) = 1710/17.6/198 ms, flip angle (FA) = 73°, sampling of 50 × 50 × 18 k-space points over 280 × 280 × 180 mm^3^, and acquisition time (TA) = 16:49 min. To obtain a water reference signal (i.e., water MRSI) with WB-MRSI, an additional dataset was obtained in an interleaved manner without water suppression and 10° excitation and gradient-echo observation (TE = 3.8 ms). A sagittal T1-weighted 3D magnetization-prepared rapid acquisition gradient-echo (MPRAGE) sequence (TR/TE/TI = 1900/2.85/900 ms, TA = 1:51 min), an axial fluid-attenuated inversion recovery (FLAIR) imaging sequence (TR/TE/TI = 12,000/115/2800 ms, TA = 1:52 min), an axial proton density-weighted imaging (PDWI) sequence (TR/TE = 4000/12 ms, TA = 1:52 min), and an axial diffusion imaging sequence (TR/TE = 4000/83.2 ms, maximum b-value = 8000 s/mm^2^, TA = 17:52 min) were also acquired to obtain structural images and to check for other abnormalities. TBI patients also received an axial 3D gradient-echo susceptibility-weighted imaging (SWI) (TR/TE = 24/3.9 ms, FA = 73°, TA = 2:21 min) to identify subtle hemorrhagic lesions such as DAI. 

### 2.3. Cognitive Function Tests

The TBI patients underwent a set of cognitive function tests, which included the Wechsler Adult Intelligence Scale (WAIS) [27], 1 s (PASAT-1s) and 2 s (PASAT-2s) paced auditory serial addition tests [28], and the trail making tests (TMT) [29]. All test results were corrected for age. 

### 2.4. Processing of WB-MRSI

#### 2.4.1. Reconstruction of Metabolite Ratio and Temperature Maps

**Figure 1:**
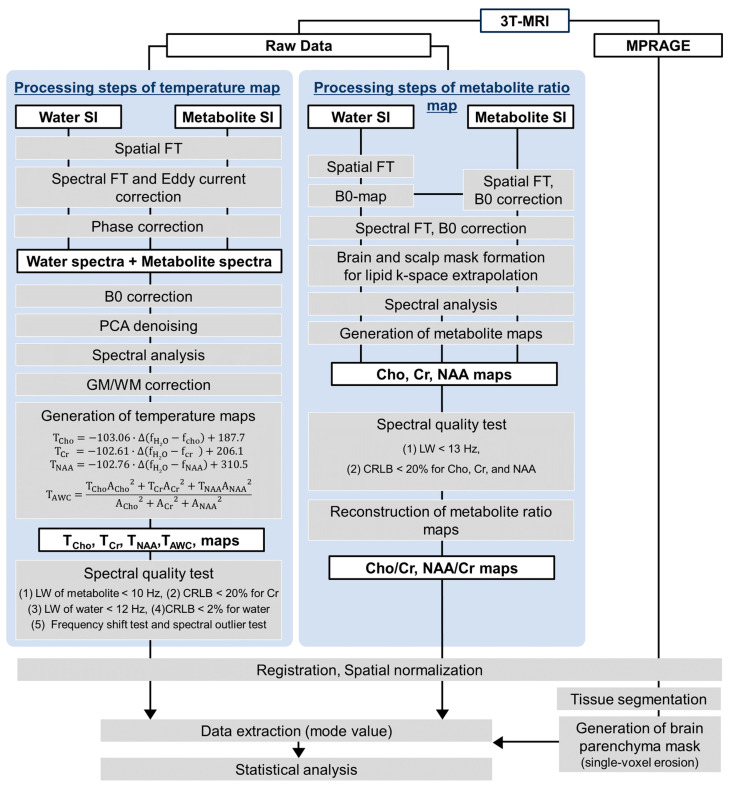
The workflow and processing steps of whole-brain magnetic resonance spectroscopic imaging (WB-MRSI). Abbreviations: SI = signal intensity; FT = Fourier transform; PCA = principal component analysis; WM = white matter; GM = gray matter; T_Cho_ = temperature derived from choline; T_Cr_ = temperature derived from creatine; T_NAA_ = temperature derived from N-acetyl aspartate; f = frequency of the respective metabolite; A = the peak amplitude for each metabolite.

WB-MRSI was processed using Metabolic Imaging and Data Analysis System (MIDAS) software version 2.35 (University of Miami, Miami, FL, USA) running on IDL version 8.4.1 (Exelis Visual Information Solutions, Boulder, CO, USA) [30]. The same procedures were applied to all participants (i.e., TBI patients and control subjects [26]). The workflow and processing steps are summarized in Figure 1. 

Since brain temperature by WB-MRSI is estimated from the frequency and amplitude of the brain’s major metabolites, this study also reconstructed the maps of major metabolite concentrations. The calculation of these concentrations (i.e., NAA, Cho, and Cr) followed the processing steps and parameters for the normal database. The steps included data resampling, spatial reconstruction, B0 correction, spatial registration, brain and scalp mask formation for lipid k-space extrapolation, spectral fitting, and signal normalization. For all metabolites, only those voxels with (1) linewidth (LW) less than 13 Hz and (2) Cramer–Rao lower bound (CRLB) less than 20% were extracted. From these metabolite concentrations, two brain metabolite ratio maps commonly used metrics in clinics, i.e., NAA/Cr and Cho/Cr, were reconstructed using ImageJ software version 1.51 (National Institutes of Health, Bethesda, MD, USA) [31]. 

The calculation of brain temperature maps followed the processing steps detailed in a previous report by Maudsley et al. [32]. The steps included spatial and spectral reconstruction, eddy current correction, phase correction, B0 correction, denoising, gray (GM) and white matter (WM) fraction correction, and temperature calculation. To avoid the inclusion of noise in the results, only those voxels with (1) LW of each metabolite less than 10 Hz, (2) CRLB of Cr less than 20%, (3) LW and CRLB of water less than 12 Hz and 2%, respectively, (4) frequency shift less than 20 Hz, and (5) voxels with values falling within mean ± 3 SD were further processed [32]. Brain temperature can be calculated from the frequencies of each major metabolite (i.e., NAA, Cho, and Cr) as well as their combination (amplitude-weighted combination) [22]. This study employed both calculations since variable performance has been reported among the methods. The brain temperature (T) derivation from the frequency (f) of each metabolite is given by the following:

(1) $$T_{Cho}=-103.06\cdot ∆\left(f_{H2O}-f_{Cho}\right)+187.7\;[{}^{\circ}C]$$

(2) $$T_{Cr}=-102.61\cdot ∆\left(f_{H2O}-f_{Cre}\right)+206.1\;[{}^{\circ}C]$$

(3) $$T_{NAA}=-102.76\cdot ∆\left(f_{H2O}-f_{NAA}\right)+310.5\;[{}^{\circ}C]$$

where T_Cho_, T_Cr_, and T_NAA_ are the temperatures derived from each metabolite concentration, f_H2O_ is the frequency of water, and f_Cho_, f_Cr_, and f_NAA_ are the frequencies of the respective metabolites. As for the brain temperature derived by amplitude-weighted combination, the temperature (T_AWC_) is given as follows:

(4) $$T_{AWC}=({T_{Cho}A_{Cho}}^{2}+{T_{Cre}A_{Cre}}^{2}+{T_{NAA}A_{NAA}}^{2})/({A_{Cho}}^{2}+{A_{Cr}}^{2}+{A_{NAA}}^{2})\;[{}^{\circ}C]$$

where A_Cho_, A_Cr_, and A_NAA_ are the peak amplitudes for each metabolite.

#### 2.4.2. Extraction of Brain Temperature and Metabolite Ratios

The temperature and metabolite ratio maps and the corresponding MPRAGE images were then spatially normalized to the standard Montreal Neurological Institute (MNI) space using the default parameters of Statistical Parametric Mapping 12 (SPM12) software (Wellcome Trust Centre for Neuroimaging, London, UK) running in MATLAB version 7.9.0 R2009b (The MathWorks, Natick, MA, USA). Voxels other than the brain tissue were removed by applying a brain parenchyma mask, which was formed by segmenting the brain parenchyma (GM and WM) voxels from the corresponding spatially normalized MPRAGE images (SPM12) and eroding a single voxel to limit partial volume errors (ImageJ). Finally, the mask was applied to the brain temperature and metabolite ratio maps, and the mode of the brain parenchyma histogram was extracted for each map. 

### 2.5. Statistical Analysis

Differences in brain temperature between TBI and control groups were compared using students’ *t*-tests or Mann–Whitney U-tests. The choice of statistical test was based on the normality of data distribution. 

The relationship between the MRI measure (i.e., mode of brain temperature or metabolite ratios) and cognitive function test scores was evaluated using partial correlation analysis in the TBI group. Our previous study on the normal database showed a variation in brain metabolite concentration with aging [26]. In addition, a considerable variation in the time lapsed from trauma was observed in the patients. Thus, correlation analysis was adjusted for age and time interval from trauma through linear regression. 

A false discovery rate (FDR)-corrected *p* < 0.05 was considered statistically significant. Statistical analysis was conducted using Statistical Package for the Social Sciences (SPSS) software version 26 (IBM, New York, NY, USA).

## 3. Results

### 3.1. Conventional MRI

**Figure 2:**
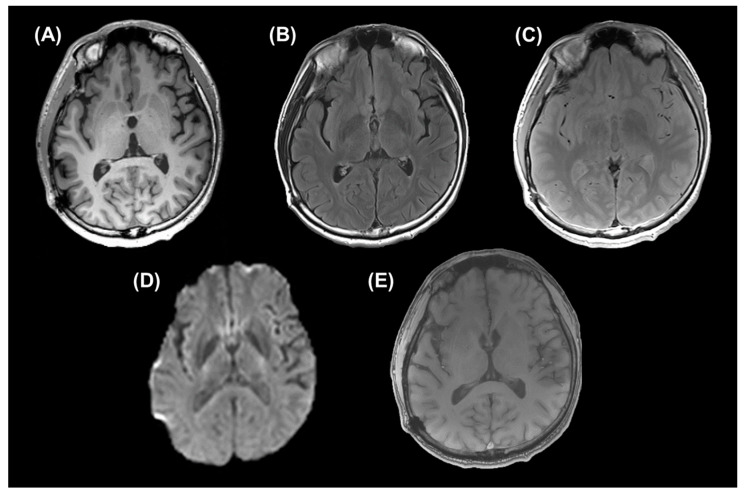
Conventional MRI sequences of a patient {48-year-old male with mild traumatic brain injury (TBI) in chronic phase}. (**A**) An axial reconstructed image of magnetization-prepared rapid acquisition gradient-echo (MPRAGE) sequence (repetition time (TR)/ echo time (TE)/ inversion time (TI) = 1900/2.85/900 ms), (**B**) an axial fluid-attenuated inversion recovery (FLAIR) image (TR/TE/TI = 12,000/115/2800 ms), (**C**) an axial proton density-weighted image (PDWI) (TR/TE = 4000/12 ms), (**D**) trace of an axial diffusion imaging sequence (TR/TE = 4000/83.2 ms, maximum b-value = 8000 s/mm^2^), and (**E**) an axial 3D gradient-echo susceptibility-weighted image (SWI) (TR/TE = 24/3.9 ms, flip angle (FA) = 73°) are given.

Six of eight patients had brain contusion or DAI on conventional MRI sequences. Representative images of a patient are given in Figure 2. 

### 3.2. Group Differences in Brain Temperature

**Figure 3:**
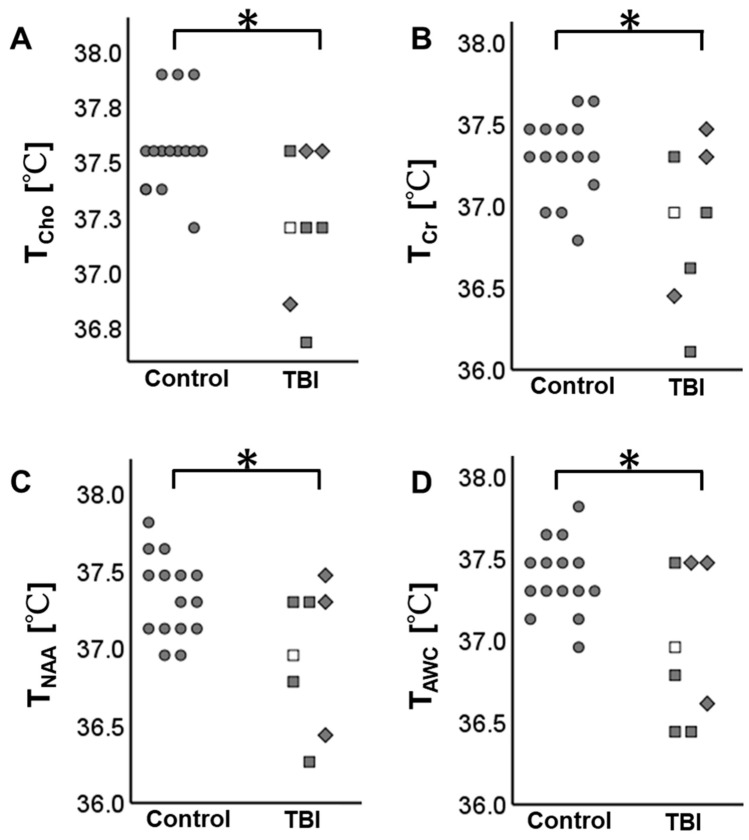
Brain temperature distribution in the participants. The TBI patients have lower brain temperature than the control subjects. (**A**) The brain temperature derived from choline (T_Cho_), (**B**) the brain temperature derived from creatine (T_Cr_), (**C**) the brain temperature derived from N-acetylaspartate (T_NAA_), and (**D**) the brain temperature derived by amplitude-weighted combination (T_AWC_). * indicates false discovery rate (FDR)-corrected *p* < 0.05. ● Control, □ mild TBI in subacute phase, ■ mild TBI in chronic phase, and ◆ moderate TBI in chronic phase.

The brain temperature measurable by any method (i.e., T_Cho_, T_Cr_, T_NAA_, or T_AWC_) was significantly lower in the TBI patients than in the control group (FDR-corrected *p* < 0.05). Figure 3 summarizes the results.

### 3.3. Relationship between Brain Temperature and Cognitive Function Test Scores

**Figure 4:**
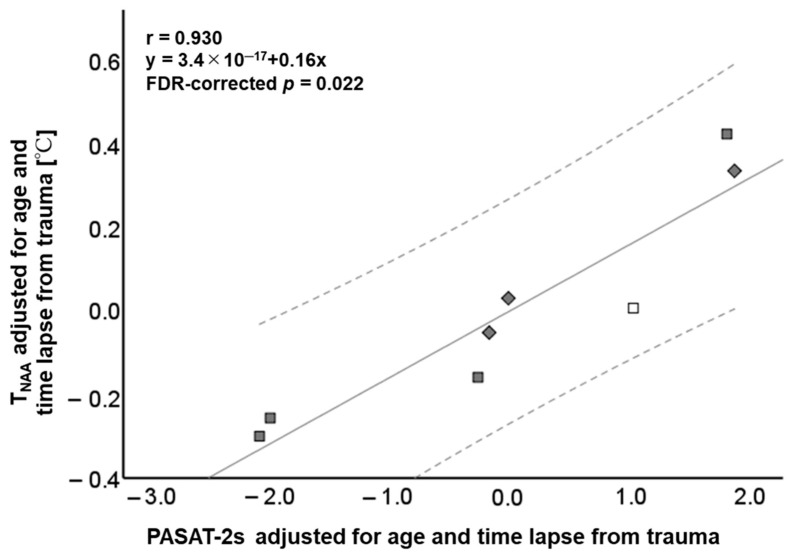
Scatterplots showing the relationship between the brain temperature derived from N-acetyl aspartate (T_NAA_) and 2 s paced auditory serial addition test (PASAT-2s) (r = 0.930, FDR-corrected *p* = 0.022). The solid line represents the mean and the dotted lines are the 95% confidence interval. □ Mild TBI in subacute phase, ■ mild TBI in chronic phase, and ◆ moderate TBI in chronic phase.

Table S1 and Figure 4 summarize significant relationships between T_NAA_ and cognitive function test scores. All variables are adjusted for age and time lapsed from trauma. A strong positive correlation was observed between T_NAA_ and PASAT-2s (r = 0.930, FDR-corrected *p* = 0.022). Brain temperature calculated by other methods (i.e., T_Cho_, T_Cr_, and T_AWC_) and the major metabolite ratios (i.e., Cho/Cr and NAA/Cr) showed no significant correlation with cognitive function test scores.

## 4. Discussion

This prospective study evaluated if the brain temperature derived noninvasively by WB-MRSI altered in TBI patients in the later phases of the disease and the potential clinical utility of brain temperature measurement. It was observed that the brain temperature decreased in the TBI patients, although they were already in the subacute or chronic phase. In addition, T_NAA_ was significantly correlated with the PASAT-2s score, which reflects the patient’s information processing ability, attention, and concentration to perform a task [28].

Our observation of lower brain temperature in TBI is consistent with a previous study that reports a decrease in lateral ventricular temperature in mild TBI patients in the subacute phase within 20 days of trauma [33]. Our study confirms their results and adds that these patients’ brain temperatures remained low until the chronic phase. A drop in the brain temperature may result from decreased metabolic activity due to glucose and oxygen depletion. In a physiological state, the chemical reaction between glucose and oxygen (glucose + 6O_2_ → 6CO_2_ + 6H_2_O) generates most of the energy required for brain metabolic activity. This reaction at 37 °C is associated with a release of ΔH° = 470 kJ of enthalpy per mol of O_2_. While some of this energy (33%) immediately dissipates into heat, the rest (67%) produces 38 adenosine triphosphate (ATP) molecules that will further maintain a complex chain of chemical reactions to ensure proper brain functioning. However, because no mechanical work is performed in the brain, the final ATP hydrolysis releases the energy back to the system, and as a result, almost all of this energy ends up as heat [34]. 

Evidence of glucose and oxygen depletion exists in TBI. According to prior reports, chronic TBI patients with a sports injury have 8–15% lower 18-fluorine fluorodeoxyglucose (^18^F-FDG) accumulation in the brain than the healthy group [35]. Similar results have been observed in Iraq war veterans with one or more episodes of mild TBI from explosive blasts [36]. In addition, one-week-post-TBI patients have been reported to experience cerebral ischemia and tissue hypoxia [37]. More than half of patients with severe TBI showed decreased cerebral blood flow (CBF) between one and six weeks after injury [38]. Furthermore, the CBF of GM is lower in moderate to severe TBI patients than in controls at six months and one year post-injury [39]. Additionally, moderate to severe TBI patients with DAI in the chronic phase had lower CBF than controls [40]. 

This study reports lower brain temperature in TBI patients in subacute to chronic phases. On the contrary, a previous report has shown increased brain temperature in acute TBI [41]. Although the cause of elevated brain temperature after brain injury is not fully understood, sepsis, inflammation, or direct injury to the hypothalamus, the body’s thermoregulatory center, might have occurred [42,43]. Alternatively, trauma-led cell membrane disruption might have induced membrane potential changes due to the excessive release of excitatory neurotransmitters [44]. In response, energy, i.e., ATP, is necessary to restore ionic equilibrium to reactivate brain cells [44,45], which further causes an increase in glucose uptake for ATP synthesis. This is consistent with the observation of an increased rate of local brain metabolism of glucose in animal models of acute TBI [46,47].

A significant correlation was observed between WB-MRSI-derived brain temperature and PASAT-2s score, suggesting the potential utility of MRI as an estimate of cognitive function test performance. Generally, cognitive function tests demand patients’ cooperation to achieve reliable results, which is not always possible in ill and pediatric patients. On the other hand, WB-MRSI for brain temperature measurement does not require any tasks. The PASAT-2s is the test in which participants answer the correct sum of numbers presented at 2 s intervals before the next number is presented [28]. The results reflect information processing ability, attention, and concentration [28]. These tests have been successfully incorporated to study the effects of TBI on information processing ability [28]. The PASAT score is considered a sensitive measure of cognitive impairment in symptomatic mild TBI patients. Previous studies have shown that approximately 54%, 38%, and 18% of mild TBI patients fall below 1.0 z, 1.5 z, and 2.0 z scores derived from norms [48]. The PASAT tests are also included in "The Minimal Assessment of Cognitive Function in MS," proposed at an international conference for the clinical monitoring of multiple sclerosis (MS), in which many patients exhibit cognitive deficits [49], and its validity has been reported [50]. Several reports have also raised concerns about the influence of brain temperature on cognitive function. For example, a study on healthy volunteers exposed to cold air has shown decreased working memory, choice reaction time, and executive function upon cold air exposure [51]. Another recent study on laboratory animals has reported the exacerbation of glymphatic drainage dysfunction in TBI by hypothermia, leading to an increase in p-tau and beta-amyloid deposition and cognitive impairment [52]. Our observation of the correlation may also imply that TBI patients have difficulties in information processing, attention, and concentration and that rehabilitation targeting these tasks may benefit them. The lack of significant correlation with other cognitive function tests may be due to the small sample size.

Of several brain temperature calculation methods, T_NAA_ performed better than the others. Similarly, it outperformed the respective metabolite ratios (i.e., NAA/Cr and Cho/Cr). Thus, T_NAA_ may become a noninvasive alternative for thermistors or an indirect indicator of the patient’s CAT performance. As a neuron marker [53], NAA has a relatively high amplitude and can easily be isolated [54]. A group of researchers has suggested that NAA is the easiest to measure accurately, giving the most reliable temperature dependence [21]. On the contrary, determinations of the chemical shifts of Cho and Cr are not so precise because Cho appears to include different components, and Cr has a low signal amplitude and is within ~0.2 ppm of Cho, resulting in peak overlap [21]. Phantom experiments have also shown a good correlation between T_NAA_ and brain temperature measured by implanted thermistor probes [21]. 

This study employed WB-MRSI as ^1^H-MRS to receive information about the brain’s metabolite concentrations and temperature. WB-MRSI is a recently developed ^1^H-MRS designed to measure the state of the brain’s major metabolites almost throughout the whole brain [30]. An echo-planar spectroscopic imaging (EPSI) sequence and short TE are coupled to enhance the acquisition time and signal-to-noise ratio. There has been another quantitative MRI technique that also permits noninvasive brain temperature measurement. This technique is diffusion imaging-based magnetic resonance (MR) thermometry and was proposed by Kozak et al. [55]. This technique estimates brain temperature from the cerebrospinal fluid (CSF) in the lateral ventricles since the diffusion coefficient of water changes with temperature. Recently, a more objective analysis method of MR thermometry based on diffusion imaging has been proposed [56,57], which has shown a significant positive correlation with the brain temperature measured by ^1^H-MRS [58]. Although this method may estimate the temperature in a shorter imaging time than WB-MRSI, it cannot directly estimate brain parenchymal temperature where water diffusion is more restricted than the CSF. Although a strong relationship between the lateral ventricular temperature measured by this method and the temperature of the right centrum semiovale measured by ^1^H-MRS has been shown, their correlation in conditions with abnormal CSF such as empyema is yet to be studied.

There are several limitations to this study. First, the sample size is small, probably due to the strict inclusion criteria set in this study. This study included only male participants because body and brain temperatures vary with the menstrual cycle in females [59,60]. Brain metabolites are also reported to fluctuate with female hormone levels [61]. Because information about the menstrual cycle could not be obtained from the database, females were excluded from the study. However, it is desired that brain temperature alterations in female TBI patients be also studied. Thus, further studies with larger sample sizes, including female patients, are needed. Also, three of eleven patients did not complete short TE WB-MRSI. Further reduction in acquisition time, achievable by incorporating machine learning technology, may help secure more patients [62]. Because of the small sample size, type II errors could have been introduced, and some important correlations might have been missed. Also, given the sample size limitation, the correlations observed in this study need to be confirmed with a larger sample size. Second, one of the patients had controlled diabetes mellitus. Four patients were on medication. The effect of diabetes mellitus and medication on brain temperature cannot wholly be excluded, although it is believed to be small.

## 5. Conclusions

Brain temperature decreases in TBI patients despite being in subacute and chronic phases of the disease. Noninvasive measurement of brain temperature in these patients may estimate the depth of brain injury responsible for impaired information processing, attention, and concentration. WB-MRSI-derived T_NAA_ may be suitable for measuring brain temperature in these patients. 

## Figures and Tables

**Table 1:** Demographic details about patients.

Patient	Age (Years)	Time from Injury (Months)	Severity of TBI	Medical and Drug History
1	20	11	Mild	Taking Lamotrigine
2	43	16	Mild	-
3	47	2	Mild	Taking traditional medicine with potential nervous system effects
4	48	68	Mild	Controlled diabetes mellitus Taking Metformin
5	68	11	Mild	-
6	33	38	Moderate to severe	-
7	39	11	Moderate to severe	Taking antihistamines
8	54	432	Moderate to severe	-

## Data Availability

The data presented in this study are available on request from the corresponding author. The data are not publicly available due to patient confidentiality or ethical restrictions.

## Supplementary Materials

The following supporting information can be downloaded at: https://www.mdpi.com/article/10.3390/metabo14010017/s1, Table S1: The relationship between MRI indices and cognitive function test scores.

## References

[1] Silverberg N.D., Iverson G.L., Cogan A., Dams-O-Connor K., Delmonico R., Graf M.J.P., Iaccarino M.A., Kajankova M., Kamins J., McCulloch K.L. (2023). The American Congress of Rehabilitation Medicine Diagnostic Criteria for Mild Traumatic Brain Injury. Arch. Phys. Med. Rehabil..

[2] MaDewan M.C., Rattani A., Gupta S., Baticulon R.E., Hung Y.C., Punchak M., Agrawal A., Adeleye A.O., Shrime M.G., Rubiano A.M. (2018). Estimating the global incidence of traumatic brain injury. J. Neurosurg..

[3] Ma V.Y., Chan L., Carruthers K.J. (2014). Incidence, prevalence, costs, and impact on disability of common conditions requiring rehabilitation in the United States: Stroke, spinal cord injury, traumatic brain injury, multiple sclerosis, osteoarthritis, rheumatoid arthritis, limb loss, and back pain. Arch. Phys. Med. Rehabil..

[4] Ghajar J. (2000). Traumatic brain injury. Lancet.

[5] Teasdale G., Jennett B. (1974). Assessment of coma and impaired consciousness. A practical scale. Lancet.

[6] Rimel R.W., Giordani B., Barth J.T., Boll T.J., Jane J.A. (1981). Disability caused by minor head injury. Neurosurgery.

[7] Rimel R.W., Giordani B., Barth J.T., Jane J.A. (1982). Moderate head injury: Completing the clinical spectrum of brain trauma. Neurosurgery.

[8] Boake C., McCauley S.R., Pedroza C., Levin H.S., Brown S.A., Brundage S.I. (2005). Lost productive work time after mild to moderate traumatic brain injury with and without hospitalization. Neurosurgery.

[9] Wintermark M., Sanelli P.C., Anzai Y., Tsiouris A.J., Whitlow C.T., ACR Head Injury Institute (2015). Imaging evidence and recommendations for traumatic brain injury: Conventional neuroimaging techniques. J. Am. Coll. Radiol..

[10] Rabinowitz A.R., Levin H.S. (2014). Cognitive sequelae of traumatic brain injury. Psychiatr. Clin. North. Am..

[11] Mott T.F., McConnon M.L., Rieger B.P. (2012). Subacute to chronic mild traumatic brain injury. Am. Fam. Physician.

[12] Barman A., Chatterjee A., Bhide R. (2016). Cognitive Impairment and Rehabilitation Strategies After Traumatic Brain Injury. Indian J. Psychol. Med..

[13] Belanger H.G., Vanderploeg R.D., Curtiss G., Warden D.L. (2007). Recent neuroimaging techniques in mild traumatic brain injury. J. Neuropsychiatry Clin. Neurosci..

[14] Le T.H., Gean A.D. (2009). Neuroimaging of traumatic brain injury. Mt. Sinai J. Med..

[15] Lee H., Wintermark M., Gean A.D., Ghajar J., Manley G.T., Mukherjee P. (2008). Focal lesions in acute mild traumatic brain injury and neurocognitive outcome: CT versus 3T MRI. J. Neurotrauma.

[16] Huisman T.A., Schwamm L.H., Schaefer P.W., Koroshetz W.J., Shetty-Alva N., Ozsunar Y., Wu O., Sorensen A.G. (2004). Diffusion tensor imaging as potential biomarker of white matter injury in diffuse axonal injury. AJNR Am. J. Neuroradiol..

[17] Grossman E.J., Ge Y., Jensen J.H., Babb J.S., Miles L., Reaume J., Silver J.M., Grossman R.I., Inglese M. (2012). Thalamus and cognitive impairment in mild traumatic brain injury: A diffusional kurtosis imaging study. J. Neurotrauma.

[18] Govindaraju V., Gauger G.E., Manley G.T., Ebel A., Meeker M., Maudsley A.A. (2004). Volumetric proton spectroscopic imaging of mild traumatic brain injury. AJNR Am. J. Neuroradiol..

[19] Friedman S.D., Brooks W.M., Jung R.E., Hart B.L., Yeo R.A. (1998). Proton MR spectroscopic findings correspond to neuropsychological function in traumatic brain injury. AJNR Am. J. Neuroradiol..

[20] Rzechorzek N.M., Thrippleton M.J., Chappell F.M., Mair G., Ercole A., Cabeleira M., Rhodes J., Marshall I., O’Neill J.S., CENTER-TBI High Resolution ICU (HR ICU) Sub-Study Participants and Investigators (2022). A daily temperature rhythm in the human brain predicts survival after brain injury. Brain.

[21] Cady E.B., D’Souza P.C., Penrice J., Lorek A. (1995). The estimation of local brain temperature by in vivo 1H magnetic resonance spectroscopy. Magn. Reson. Med..

[22] Cady E.B., Penrice J., Robertson N.J. (2011). Improved reproducibility of MRS regional brain thermometry by ‘amplitude-weighted combination’. NMR Biomed..

[23] Corbett R.J., Laptook A.R., Tollefsbol G., Kim B. (1995). Validation of a noninvasive method to measure brain temperature in vivo using 1H NMR spectroscopy. J. Neurochem..

[24] Kuroda K., Takei N., Mulkern R.V., Oshio K., Nakai T., Okada T., Matsumura A., Yanaka K., Hynynen K., Jolesz F.A. (2003). Feasibility of internally referenced brain temperature imaging with a metabolite signal. Magn. Reson. Med. Sci..

[25] Mueller C., Lin J.C., Sheriff S., Maudsley A.A., Younger J.W. (2020). Evidence of widespread metabolite abnormalities in Myalgic encephalomyelitis/chronic fatigue syndrome: Assessment with whole-brain magnetic resonance spectroscopy. Brain Imaging Behav..

[26] Li X., Abiko K., Sheriff S., Maudsley A.A., Urushibata Y., Ahn S., Tha K.K. (2022). The Distribution of Major Brain Metabolites in Normal Adults: Short Echo Time Whole-Brain MR Spectroscopic Imaging Findings. Metabolites.

[27] Lichtenberger E.O., Kaufman A.S. (2012). Essentials of WAIS-IV Assessment.

[28] Tombaugh T.N. (2006). A comprehensive review of the Paced Auditory Serial Addition Test (PASAT). Arch. Clin. Neuropsychol..

[29] Reitan R.M. (1958). Validity of the Trail Making Test as an Indicator of Organic Brain Damage. Percept. Mot. Skills.

[30] Maudsley A.A., Darkazanli A., Alger J.R., Hall L.O., Schuff N., Studholme C., Yu Y., Ebel A., Frew A., Goldgof D. (2006). Comprehensive processing, display and analysis for in vivo MR spectroscopic imaging. NMR Biomed..

[31] Schneider C.A., Rasband W.S., Eliceiri K.W. (2012). NIH Image to ImageJ: 25 years of image analysis. Nat. Methods.

[32] Maudsley A.A., Goryawala M.Z., Sheriff S. (2017). Effects of tissue susceptibility on brain temperature mapping. Neuroimage.

[33] Tazoe J., Yamada K., Sakai K., Akazawa K., Mineura K. (2014). Brain core temperature of patients with mild traumatic brain injury as assessed by DWI-thermometry. Neuroradiology.

[34] Yablonskiy D.A., Ackerman J.J., Raichle M.E. (2000). Coupling between changes in human brain temperature and oxidative metabolism during prolonged visual stimulation. Proc. Natl. Acad. Sci. USA.

[35] Provenzano F.A., Jordan B., Tikofsky R.S., Saxena C., Van Heertum R.L., Ichise M. (2010). F-18 FDG PET imaging of chronic traumatic brain injury in boxers: A statistical parametric analysis. Nucl. Med. Commun..

[36] Peskind E.R., Petrie E.C., Cross D.J., Pagulayan K., McCraw K., Hoff D., Hart K., Yu C.E., Raskind M.A., Cook D.G. (2011). Cerebrocerebellar hypometabolism associated with repetitive blast exposure mild traumatic brain injury in 12 Iraq war Veterans with persistent post-concussive symptoms. Neuroimage.

[37] Veenith T.V., Carter E.L., Geeraerts T., Grossac J., Newcombe V.F., Outtrim J., Gee G.S., Lupson V., Smith R., Aigbirhio F.I. (2016). Pathophysiologic Mechanisms of Cerebral Ischemia and Diffusion Hypoxia in Traumatic Brain Injury. JAMA Neurol..

[38] Inoue Y., Shiozaki T., Tasaki O., Hayakata T., Ikegawa H., Yoshiya K., Fujinaka T., Tanaka H., Shimazu T., Sugimoto H. (2005). Changes in cerebral blood flow from the acute to the chronic phase of severe head injury. J. Neurotrauma.

[39] Gaggi N.L., Ware J.B., Dolui S., Brennan D., Torrellas J., Wang Z., Whyte J., Diaz-Arrastia R., Kim J.J. (2023). Temporal dynamics of cerebral blood flow during the first year after moderate-severe traumatic brain injury: A longitudinal perfusion MRI study. Neuroimage Clin..

[40] Kim J., Whyte J., Patel S., Avants B., Europa E., Wang J., Slattery J., Gee J.C., Coslett H.B., Detre J.A. (2010). Resting cerebral blood flow alterations in chronic traumatic brain injury: An arterial spin labeling perfusion FMRI study. J. Neurotrauma.

[41] Pegoli M., Zurlo Z., Bilotta F. (2020). Temperature management in acute brain injury: A systematic review of clinical evidence. Clin. Neurol. Neurosurg..

[42] Badjatia N. (2009). Hyperthermia and fever control in brain injury. Crit. Care Med..

[43] Thompson H.J., Tkacs N.C., Saatman K.E., Raghupathi R., McIntosh T.K. (2003). Hyperthermia following traumatic brain injury: A critical evaluation. Neurobiol. Dis..

[44] Werner C., Engelhard K. (2007). Pathophysiology of traumatic brain injury. Br. J. Anaesth..

[45] Prins M., Greco T., Alexander D., Giza C.C. (2013). The pathophysiology of traumatic brain injury at a glance. Dis. Model. Mech..

[46] Yoshino A., Hovda D.A., Kawamata T., Katayama Y., Becker D.P. (1991). Dynamic changes in local cerebral glucose utilization following cerebral conclusion in rats: Evidence of a hyper- and subsequent hypometabolic state. Brain Res..

[47] Kawamata T., Katayama Y., Hovda D.A., Yoshino A., Becker D.P. (1992). Administration of excitatory amino acid antagonists via microdialysis attenuates the increase in glucose utilization seen following concussive brain injury. J. Cereb. Blood Flow. Metab..

[48] Cicerone K.D. (1997). Clinical sensitivity of four measures of attention to mild traumatic brain injury. Clin. Neuropsychol..

[49] Benedict R.H., Fischer J.S., Archibald C.J., Arnett P.A., Beatty W.W., Bobholz J., Chelune G.J., Fisk J.D., Langdon D.W., Caruso L. (2002). Minimal neuropsychological assessment of MS patients: A consensus approach. Clin. Neuropsychol..

[50] Benedict R.H., Cookfair D., Gavett R., Gunther M., Munschauer F., Garg N., Weinstock-Guttman B. (2006). Validity of the minimal assessment of cognitive function in multiple sclerosis (MACFIMS). J. Int. Neuropsychol. Soc..

[51] Muller M.D., Gunstad J., Alosco M.L., Miller L.A., Updegraff J., Spitznagel M.B., Glickman E.L. (2012). Acute cold exposure and cognitive function: Evidence for sustained impairment. Ergonomics.

[52] Gu W., Bai Y., Cai J., Mi H., Bao Y., Zhao X., Lu C., Zhang F., Li Y.H., Lu Q. (2023). Hypothermia impairs glymphatic drainage in traumatic brain injury as assessed by dynamic contrast-enhanced MRI with intrathecal contrast. Front. Neurosci..

[53] Rae C.D. (2014). A guide to the metabolic pathways and function of metabolites observed in human brain 1H magnetic resonance spectra. Neurochem. Res..

[54] Corbett R., Tollefsbol G., Laptook A. Measurement of brain temperature in vivo using NMR spectroscopy. Proceedings of the SMR, 2nd Annual Meeting.

[55] Kozak L.R., Bango M., Szabo M., Rudas G., Vidnyanszky Z., Nagy Z. (2010). Using diffusion MRI for measuring the temperature of cerebrospinal fluid within the lateral ventricles. Acta Paediatr..

[56] Sakai K., Yamada K., Mori S., Sugimoto N., Nishimura T. (2011). Age-dependent brain temperature decline assessed by diffusion-weighted imaging thermometry. NMR Biomed..

[57] Sakai K., Yamada K., Sugimoto N. (2012). Calculation methods for ventricular diffusion-weighted imaging thermometry: Phantom and volunteer studies. NMR Biomed..

[58] Sumida K., Sato N., Ota M., Sakai K., Sone D., Yokoyama K., Kimura Y., Maikusa N., Imabayashi E., Matsuda H. (2016). Intraventricular temperature measured by diffusion-weighted imaging compared with brain parenchymal temperature measured by MRS in vivo. NMR Biomed..

[59] Kelly G. (2006). Body temperature variability (Part 1): A review of the history of body temperature and its variability due to site selection, biological rhythms, fitness, and aging. Altern. Med. Rev..

[60] Tsukamoto T., Shimono T., Sai A., Sakai K., Yamamoto A., Sakamoto S., Miki Y. (2016). Assessment of brain temperatures during different phases of the menstrual cycle using diffusion-weighted imaging thermometry. Jpn. J. Radiol..

[61] Rasgon N.L., Thomas M.A., Guze B.H., Fairbanks L.A., Yue K., Curran J.G., Rapkin A.J. (2001). Menstrual cycle-related brain metabolite changes using 1H magnetic resonance spectroscopy in premenopausal women: A pilot study. Psychiatry Res..

[62] Lam F., Li Y., Guo R., Clifford B., Peng X., Liang Z.P. (2018). Further accelerating SPICE for ultrafast MRSI using learned spectral features. Proc. Intl. Soc. Mag. Reson. Med..

