# Supplementary material for: Brain Temperature as an Indicator of Cognitive Function in Traumatic Brain Injury Patients
Source: Metabolites. 2023 Dec 27;14(1):17. doi: 10.3390/metabo14010017 (PMC10818445; doi:10.3390/metabo14010017)
Supplement: Supplementary file 1 [file metabolites-14-00017-s001.zip › metabolites-2722684-supplementary.pdf]

Table S1. The relationship between MRI measures and cognitive function test scores

		WAIS										
		Verbal IQ	Performance IQ	Full scale IQ	Verbal comprehension	perceptual organization	Working memory	Processing speed	PASAT-2s	PASAT-1s	TMT-A	TMT-B
T_{Cho}	Correlation	0.435	0.490	0.495	0.492	0.223	0.333	0.646	0.795	0.849	-0.144	-0.445
	P-value (FDR-corrected P-value)	0.329 (0.452)	0.264 (0.452)	0.259 (0.452)	0.262 (0.452)	0.630 (0.693)	0.466 (0.570)	0.117 (0.429)	0.032 (0.176)	0.016 (0.176)	0.758 (0.758)	0.317 (0.452)
T_{Cre}	Correlation	0.636	0.768	0.743	0.603	0.558	0.609	0.872	0.890	0.778	-0.460	-0.726
	P-value (FDR-corrected P-value)	0.125 (0.186)	0.044 (0.117)	0.056 (0.117)	0.152 (0.186)	0.193 (0.212)	0.147 (0.186)	0.011 (0.061)	0.007 (0.061)	0.039 (0.117)	0.299 (0.299)	0.064 (0.117)
T_{NAA}	Correlation	0.677	0.709	0.734	0.733	0.429	0.512	0.810	0.930	0.793	-0.429	-0.646
	P-value (FDR-corrected P-value)	0.095 (0.149)	0.075 (0.138)	0.060 (0.134)	0.061 (0.134)	0.337 (0.337)	0.240 (0.293)	0.027 (0.121)	0.002 (0.022)	0.033 (0.121)	0.336 (0.337)	0.117 (0.161)
T_{AWC}	Correlation	0.447	0.540	0.525	0.473	0.294	0.403	0.702	0.806	0.767	-0.235	-0.530
	P-value (FDR-corrected P-value)	0.315 (0.433)	0.211 (0.414)	0.226 (0.414)	0.284 (0.433)	0.523 (0.575)	0.370 (0.452)	0.079 (0.290)	0.029 (0.242)	0.044 (0.242)	0.612 (0.612)	0.221 (0.414)
ChoCr	Correlation	-0.497	-0.459	-0.495	-0.585	-0.324	-0.245	-0.394	-0.437	-0.032	0.646	0.461
	P-value (FDR-corrected P-value)	0.257 (0.514)	0.300 (0.514)	0.259 (0.514)	0.167 (0.514)	0.479 (0.585)	0.596 (0.656)	0.382 (0.525)	0.327 (0.514)	0.946 (0.946)	0.117 (0.514)	0.298 (0.514)
NAACr	Correlation	0.193	0.171	0.213	0.114	0.088	0.242	0.278	0.297	0.787	0.358	0.000
	P-value (FDR-corrected P-value)	0.678 (0.936)	0.714 (0.936)	0.647 (0.936)	0.807 (0.936)	0.851 (0.936)	0.601 (0.936)	0.546 (0.936)	0.518 (0.936)	0.036 (0.396)	0.431 (0.936)	1.000 (1.000)

p and r values for correlation tests between MRI measures and cognitive function test scores after adjusting for age and time lapsed from injury. Abbreviation: T_{Cho} = brain temperature derived from choline; T_{Cr} = brain temperature derived from creatine; T_{NAA} = brain temperature derived from N-acetylaspartate; T_{AWC} = brain temperature derived by amplitude-weighted combination; WAIS = Wechsler Adult Intelligence Scale; PASAT-2s = 2 s paced auditory serial addition test; PASAT-1s = 1 s paced auditory serial addition test; TMT = trail making test.
